# Outcomes of Patients with Benign Liver Diseases Undergoing Living Donor versus Deceased Donor Liver Transplantation

**DOI:** 10.1371/journal.pone.0027366

**Published:** 2011-11-07

**Authors:** Chuan Li, Kai Mi, Tian fu Wen, Lu nan Yan, Bo Li, Jia ying Yang, Ming qing Xu, Wen tao Wang, Yong gang Wei

**Affiliations:** Division of Liver Transplantation, West China Hospital, Sichuan University, Chengdu, Sichuan Province, China; The University of Hong Kong, Hong Kong

## Abstract

**Background/Aims:**

The number of people undergoing living donor liver transplantation (LDLT) has increased rapidly in many transplant centres. Patients considering LDLT need to know whether LDLT is riskier than deceased donor liver transplantation (DDLT). The aim of this study was to compare the outcomes of patients undergoing LDLT versus DDLT.

**Methods:**

A total of 349 patients with benign liver diseases were recruited from 2005 to 2011 for this study. LDLT was performed in 128 patients, and DDLT was performed in 221 patients. Pre- and intra-operative variables for the two groups were compared. Statistically analysed post-operative outcomes include the postoperative incidence of complication, biliary and vascular complication, hepatitis B virus (HBV) recurrence, long-term survival rate and outcomes of emergency transplantation.

**Results:**

The waiting times were 22.10±15.31 days for the patients undergoing LDLT versus 35.81±29.18 days for the patients undergoing DDLT. The cold ischemia time (CIT) was 119.34±19.75 minutes for the LDLT group and 346±154.18 for DDLT group. LDLT group had higher intraoperative blood loss, but red blood cell (RBC) transfusion was not different. Similar ≥ Clavien III complications, vascular complications, hepatitis B virus (HBV) recurrence and long-term survival rates were noted. LDLT patients suffered a higher incidence of biliary complications in the early postoperative days. However, during the long-term follow-up period, biliary complication rates were similar between the two groups. The long-term survival rate of patients undergoing emergency transplantation was lower than of patients undergoing elective transplantation. However, no significant difference was observed between emergency LDLT and emergency DDLT.

**Conclusions:**

Patients undergoing LDLT achieved similar outcomes to patients undergoing DDLT. Although LDLT patients may suffer a higher incidence of early biliary complications, the total biliary complication rate was similar during the long-term follow-up period.

## Introduction

Liver transplantation is perceived as the only curative treatment for patients with end-stage liver disease. However, worldwide scarcity of deceased donor liver grafts continues to be a limitation of this management. Approximately 20–25% of patients with liver failure die while waiting for a liver transplant, and another 20–30% of patients with hepatocellular carcinoma drop off the waiting list because of tumour progression.[1], [2], [3], [4] Since Strong et al.[5] performed the first successful LDLT in 1989, LDLT has emerged as the alternative life-saving treatment to DDLT. Over the past 2 decades, the number of LDLTs has steadily increased in many transplant centres, especially in Asia.[6], [7] LDLT has the following advantages over DDLT: a shorter wait time, a shorter cold ischemic time, and a better organisation of the surgery time.[8] However, donor risks are inevitable and are an undeniable problem that troubles transplant surgeons. Moreover, LDLT has a smaller biliary and vascular calibre and an additional transection step, which may potentially increase the surgical risk and the incidence of postoperative complications. Previous investigations have suggested that patients undergoing LDLT may have a higher incidence of biliary and vascular complications and a lower long-term survival rate than patients undergoing DDLT.[9], [10] As surgical techniques and postoperative managements continue to advance, the outcomes of LDLT have continued to improve. Patients considering LDLT should know whether the risk, severity of complications and long-term survival are similar to DDLT. However, few reports exist in the literature addressing these topics.

The LDLT program at our centre was initiated in 2001. Because of the scarcity of cadaveric donor, the number of LDLTs continuously increased at our transplant centre. Similar to other transplant centres, the principle concern is donor safety, and we have done our best to examine the lessons learned from previous transplantations.[11] Clarifying the mortality and long-term survival of LDLT and DDLT is important to recipients and donors. Thus, we conducted this study to investigate whether the outcomes for LDLT versus DDLT are different.

## Methods

### Study group

Adult patients with benign end-stage liver diseases who received DDLT or adult-to-adult right hepatic lobe LDLT without middle hepatic vein from 2005 to 2011 were analysed in the current study. Patients who underwent the following procedures were excluded from the study: dual grafts liver transplantation, split liver transplantation, retransplantation, ABO incompatible transplantation and combined liver and kidney transplantation. According to the procedure the patients underwent, two groups were organised into patients who underwent the LDLT procedure versus the DDLT procedure. All clinical investigations were conducted following the principles expressed in the declaration of Helsinki. The identities of all of the study subjects were anonymous and were kept confidential. The current study and the transplant operations were approved by the ethics committee of the West China Hospital, Sichuan University. The ethics committee also approved the retrospective analysis of existing patient data without informed consent because of the low risk for breaching confidentiality.

### Donor selection

Donors must be ABO blood type compatible and have negative laboratory findings. For LDLT, donors must be close relatives. Volumetric computed tomography with contrast was administrated to evaluate the right hepatic lobe of all donors. The right hepatic lobe of donors without middle hepatic vein must be at least 0.8% of the recipient's standard weight and the remaining liver remnant in the donor must be at least 40%.[11]

### Surgical procedure

A detail technical description of the surgical procedure was described in previous investigations.[11] Briefly, the surgery was performed through a right subcostal incision with an extension to the upper midline after general anaesthesia. A liver biopsy was used to evaluate the amount of steatosis present. An intraoperative cholangiogram via the cystic duct was performed to assess the biliary anatomy after the cholecystectomy. Liver transection was performed using CUSA™ without inflow occlusion. All hepatic veins greater than 0.5 cm, including the short hepatic vein and the distributions of the MHV, were preserved at the time of harvesting for potential anastomoses in the recipient. At the end of the liver transection, the right hepatic artery, bile duct and portal vein were divided.

All grafts were preserved and flushed using the University of Wisconsin solution in both the LDLT and DDLT groups. The hepatic artery reconstruction was performed after reperfusion. If the condition of the recipient's hepatic artery was inadequate, a jump graft to the aorta was constructed using the recipient's saphenous vein. For bile duct construction, duct-to-duct anastomosis was the first choice. A bile duct stent was not used in all cases. If the recipient's bile duct was inadequate, a Rou-en-Y hepaticojejunostomy was performed. A venous-venous bypass was not utilised in either the LDLT or DDLT groups.

### Emergency liver transplantation

No national criteria for emergency liver transplantation in mainland China exist. In our centre's province, priorities for an emergency liver transplantation are as follows: hepatic encephalopathy ≥ grade III or prothrombin time activity less than 20% or irreversible complications such as severe upper gastrointestinal bleeding, or hepatorenal syndrome.[12]

### Immunosuppression regimen and antivirus protocols

A calcineurin inhibitor (CNI, tacrolimus or cyclosporine) agent, mycophenolate mofetil and steroid were used to maintain immunosuppression. Steroid pulse therapy was conducted in patients with rejection. Steroid therapy was tapered off rapidly whenever possible. Lamivudine and hepatitis B immune globulin was administered to prevent HBV recurrence for HBsAg positive patients after transplantation. Moreover, hepatitis B immune globulin was given to HBV patients during transplantation. HBV recurrence after liver transplantation was defined as the reappearance of HBsAg or HBV DNA in the serum.[13] For patients with recurrent HBV, tests to determine the viral mutation against Lamivudine were performed. New anti-viral therapy was administrated to patients with recurrent HBV based on the result of the viral mutation against Lamivudine. The addition of Adefovir or replacement with Entecavir was carried out based on the mutation locus.

### Follow-up and data collection

The following preoperative and intraoperative data for both groups were collected: age, gender, body mass index (BMI), model for end-stage liver disease (MELD) score, child-pugh status, aetiology for transplantation, graft to recipient weight ratio (GRWR), total bilirubin (TB), creatinine (Crea) level, international normalised ratio (INR), waiting time, cold ischemic time (CIT), intraoperative blood loss and red blood cell (RBC) transfusion. Postoperative complications were classified using the Clavien-Dindo classification system.[14], [15] An early postoperative complication was defined as a complication occurring within 3 months after transplantation.[16] Complications occurring greater than 3 months were considered late complications.[16] Renal dysfunction was defined as a creatinine level greater than 1.5 mg/dL.[17] CIT was defined as the time between the cross-clamping of donor vessels and portal reperfusion in the recipient.[18]

### Statistical analysis

Continuous variables were presented as the mean ± SD. Categorical variables were analysed using the chi-square test or Fisher's exact test, whereas one-way analysis of variance was used to analyse continuous variables. The Kaplan-Meier method with log-rank test was utilised to compare the long-term survival of the two groups. A *p* value of less than 0.05 was considered as statistically significant.

## Results

### Demographics of the two groups

A total of 349 patients were recruited for the present study, including 128 LDLT recipients and 221 DDLT recipients. The mean follow-up periods for the LDLT and DDLT groups were 34.58±21.53 months and 45.68±25.90 months respectively. The primary causes for transplantation were HBV related diseases in both groups. In the LDLT group, 11 patients were intensive care unit (ICU) bound, whereas in the DDLT group, 13 patients were ICU bound (*P* = 0.335). Table 1 presents the univariable analysis comparing the LDLT and DDLT groups. There was no significant in age, gender, aetiology, MELD score, starting TB, ALB, Crea and INR level, and so forth between the two groups.

**Table 1:** Preoperative and intraoperative characteristics of the LDLT and DDLT groups.

Variables	LDLT	DDLT	*P*
**Donor variables**			
Age (year)	33.53±9.08	32.81±7.34	0.422
Gender (female)	35	43	0.088
BMI	22.71±2.42	22.46±1.46	0.206
**Recipient variables**			
Age (year)	42.96±8.57	44.55±9.71	0.126
Gender (female)	20	42	0.470
BMI	22.36±3.18	22.03±3.02	0.342
MELD score	19.55±10.69	18.19±9.63	0.223
Child-Pugh score	8.57±2.29	8.31±2.55	0.337
Starting TB level (µmol/L)	161.28±198.77	135.96±185.21	0.232
Starting Crea level (µmol/L)	88.9±55.52	86.02±55.23	0.635
Starting INR level	1.97±1.70	1.75±1.06	0.144
Starting HB level (g/L)	115.17±22.95	111.16±21.96	0.106
ICU bound	11	13	0.335
**Graft variables**			
GRWR	0.96±0.02%	/	/
**Surgical variables**			
CIT	119.34±19.75	346±154.18	<0.001
Blood loss	2191.02±189.24	1733.94±82.15	0.011
RBC transfusion	4.70±5.38	3.84±6.35	0.200
**Comorbidities**			
Blood hypertension	3	8	0.752
Diabetes	2	7	0.459
Pretransplantation dialysis	2	0	0.134
Upper abdominal surgery history	25	56	0.215
**Aetiology**			0.105
HBV	113	208	
Alcoholic liver cirrhosis	2	5	
Hepatolithiasis	4	1	
Autoimmune hepatitis	1	4	
HCV	3	1	
hepatic hydatidosis	2	1	
primary biliary cirrhosis	0	1	
trauma	1	0	
huge hepatic hemangioma	1	0	
polycystic liver	1	0	
**Waiting time**	22.10±15.31	35.81±29.18	<0.001

### Waiting time and intraoperative characteristics of the two groups

When we compared the time between that patients enrolled in the waiting list and the transplant time of the two groups, the waiting time of the LDLT group was only 22.10±15.31 days (range: 3 days to 86 days), which was significantly shorter than for the DDLT group (35.81±29.18 days (range: 6 days to 197 days)) (*P*<0.001).

CIT of the two groups was 119.34±19.75 minutes for the LDLT group and 346±154.18 minutes for the DDLT group (*P*<0.001). During the operation, the mean estimated blood loss was 2191.02±189.24 mL in the LDLT group and 1733.94±82.15 mL in the DDLT group (*P* = 0.011), respectively. When comparing the units of allogenic RBC transfusions, no significant difference was observed between the two groups (4.70±5.38 units for LDLT versus 3.84±6.35 units for DDLT, *P* = 0.200).

### Severe early complications between the two groups

According to the Clavien-Dindo classification, the grade I and grade II complications are trivial and not life-threatening.[19] We thus compared the number of patients who experienced early complications ≥ Clavien III between the two groups. Severe early complications occurred in 74 of the 349 recipients (table 2) within 3 months after transplantation. The incidence of severe early complications of the LDLT group was higher than the DDLT group (23.44% versus 19.91%). However, this difference did not reach significance (*P* = 0.387). A total of 34 (9.74%) patients died within 3 months after transplantation. The causes included multiple organ dysfunction syndrome (MODS, n = 15), infection (n = 12), renal dysfunction (n = 3), hepatic arterial thrombosis (HAT, n = 1), rejection (n = 1), intracranial haemorrhage (n = 1) and graft versus host disease (GVHD, n = 1). Two recipients were readmitted to the ICU for severe respiratory failure. One patient suffered from renal dysfunction.

**Table 2:** Severe early postoperative complications of the LDLT and DDLT groups.

Complications	LDLT	DDLT	*P*
**Grade IIIa**			
Thoracentesis for pleural effusion	4	7	
Abdominocentesis	1	3	
ENBD for bile leak	2	0	
**Grade IIIb**			
Reoperation for bleeding	1	2	
Reoperation for bile leak	1	1	
Reoperation for portal vein thrombosis	0	1	
Reoperation for abdominal infection	0	1	
Reoperation for biliary stone	0	1	
hepatic arterial complications	1	1	
Intervention for portal vein stricture	1	2	
Intervention for IVC stricture	0	1	
Intervention for biliary stricture	1	5	
**Grade IV**			
Renal dysfunction	0	1	
Return to ICU for respiratory failure	1	1	
**Grade V**	17	17	
Total	30(23.44%)	44(19.91%)	0.387

### Biliary problems between the two groups

As listed in table 3, 43 (12.32%) of the 349 patients had variable biliary complications, excluding of 3 cases in which biliary problems were secondary to vascular complications. In the LDLT group, 19 (14.84%) recipients suffered from biliary complications, whereas 24 (10.86%) DDLT recipients had variable biliary problems. The incidences of total biliary complications between the two groups during the long-term follow-up, including early and late biliary complications, were similar (*P* = 0.275). When we compared the early biliary complications (≤ 3 months) between the two groups, the LDLT group showed a higher incidence of biliary complications (table 3, *P* = 0.023). The most common biliary complication within the first 3 months after transplantation was a bile leak in the LDLT group and biliary stricture in the DDLT group. A total of 15 patients suffered from bile leaks. Among these patients, only 6 patients received special interventions, including reoperations for 3 patients, endoscopic nasobiliary drainage (ENBD) for 2 patients and abdominocentesis for 1 patient. All patients with a bile duct stone underwent a reoperation. Endoscopic surgical procedures, such as stent, sphincterotomy and balloon dilation, were performed on 16 patients with biliary strictures. Additionally, another 6 patients with biliary strictures underwent a reoperation.

**Table 3:** Postoperative biliary complications of the LDLT and DDLT groups.

	Early biliary complications			Total biliary complications		
	LDLT	DDLT	*P*	LDLT	DDLT	*P*
Bile leak	12	3		12	3	
Stricture	2	6		7	15	
Stone	0	1		0	6	
Total	14(10.94%)	10(4.52%)	0.023	19(14.84%)	24(10.86%)	0.275

### Vascular complications

As presented in table 4, 9 (2.58%) of the 349 recipients had vascular complications during the follow-up period. Hepatic arterial complications were observed in 3 patients, including HAT in 1 (0.78%) LDLT recipient and 1 (0.45%) DDLT recipient and hepatic artery stenosis in 1 (0.78%) LDLT recipient. The 2 HAT patients died, whereas the patient with hepatic artery stenosis survived after undergoing a balloon dilation and stent placement procedure. One (0.45%) DDLT recipient suffered an inferior vena cava (IVC) stenosis and underwent a balloon dilation procedure. Portal vein stenosis was observed in 2 (1.49%) LDLT recipients and 2 (0.89%) DDLT recipients. The 4 portal vein stenosis patients recovered after a balloon dilation procedure. One (0.45%) DDLT recipient underwent a reoperation for portal vein thrombosis. One (0.78%) LDLT recipient underwent a balloon dilation procedure for hepatic vein stenosis.

**Table 4:** Postoperative vascular complications of the LDLT and DDLT groups.

Vascular complications	LDLT	DDLT	Treatments
HAT	1	1	Died
Hepatic artery stenosis	0	1	Balloon dilation + stent
PVT	0	1	Reoperation
Portal vein stenosis	2	2	Balloon dilation
IVC stenosis	0	1	Balloon dilation
Hepatic vein stenosis	1	0	Balloon dilation

### Laboratory test changes after transplantation

**Figure 1:**
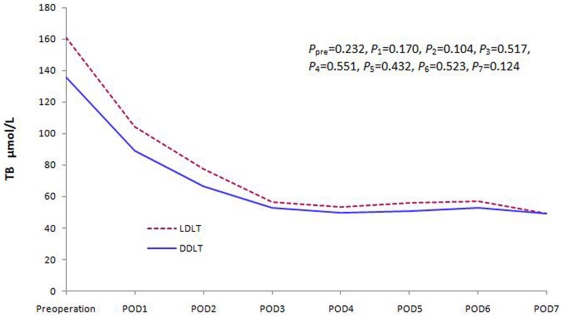
TB level changes during the first postoperative week (*P*_pre_ *= *0.232, *P*_1_ = 0.170, *P*_2_ = 0.104, *P*_3_ = 0.517, *P*_4_ = 0.551, *P*_5_ = 0.432, *P*_6_ = 0.523, *P*_7_ = 0.124).

**Figure 2:**
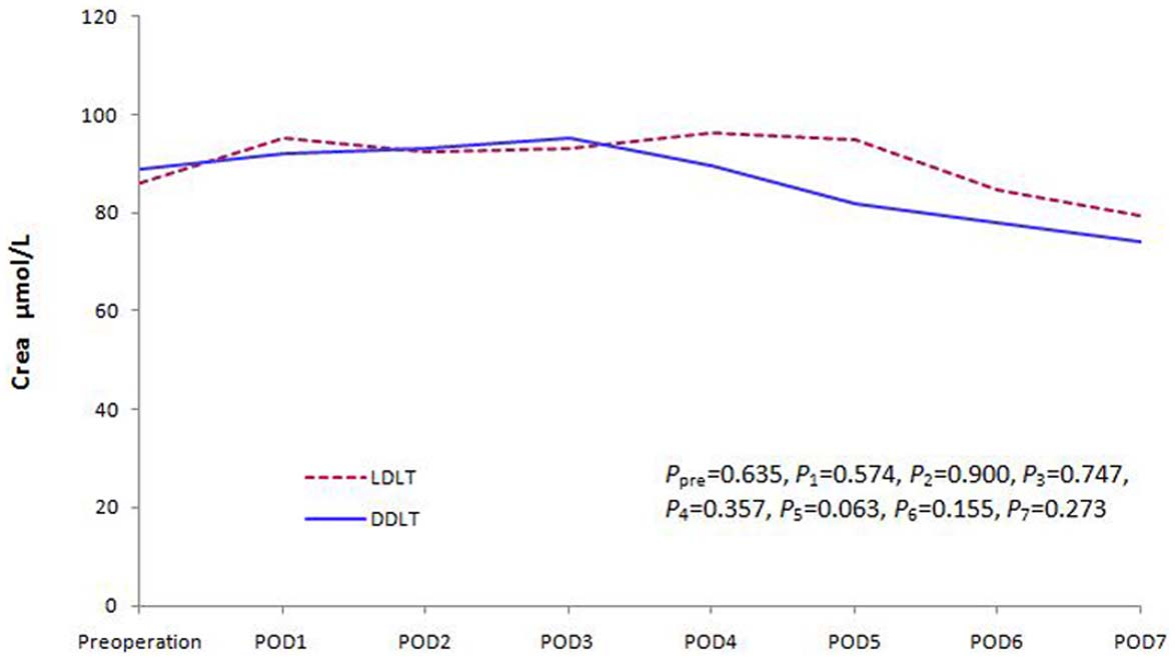
Creatinine level changes during the first postoperative week (*P*_pre_ *= *0.635, *P*_1_ = 0.574, *P*_2_ = 0.900, *P*_3_ = 0.747, *P*_4_ = 0.357, *P*_5_ = 0.063, *P*_6_ = 0.155, *P*_7_ = 0.273).

**Figure 3:**
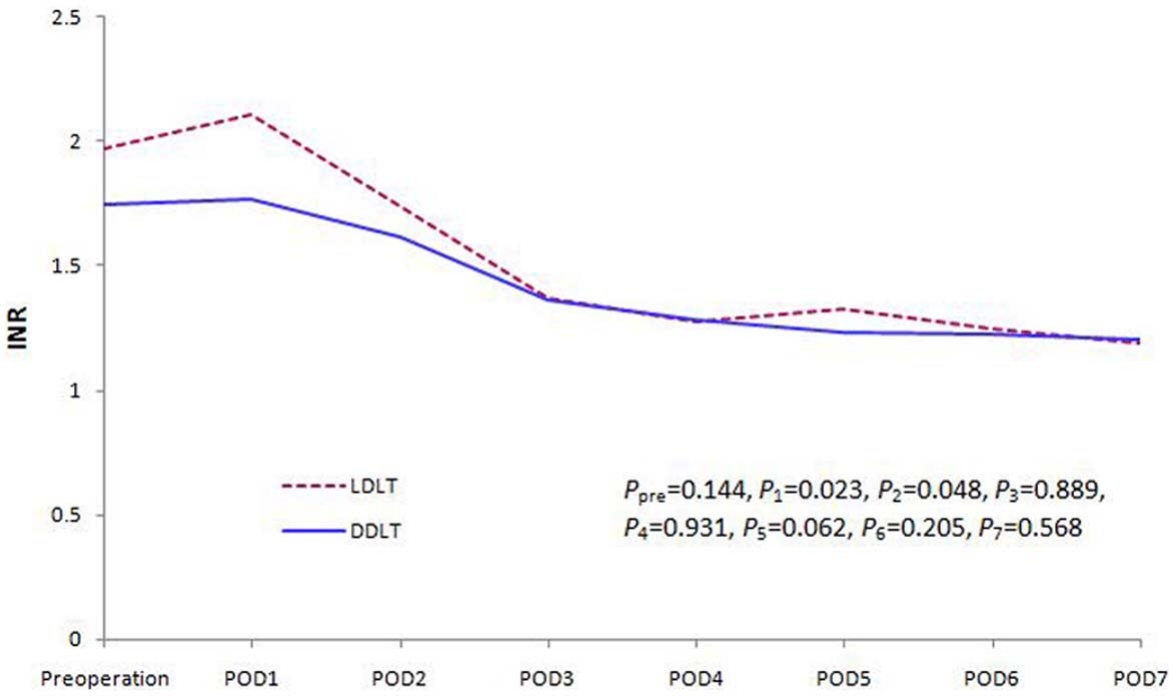
INR level changes during the first postoperative week (*P*_pre_ *= *0.144, *P*_1_ = 0.023, *P*_2_ = 0.048, *P*_3_ = 0.889, *P*_4_ = 0.931, *P*_5_ = 0.062, *P*_6_ = 0.205, *P*_7_ = 0.568).

We compared liver, renal and coagulation function changes between the two groups. There was no significant difference in the recovery of the liver and renal function within the first postoperative week (Figure 1 and 2). However, the coagulation function of the patients in the LDLT group was worse than DDLT group during the early postoperative days (POD). This difference disappeared soon during the later follow-up period (Figure 3).

### HBV recurrence of two groups

**Figure 4:**
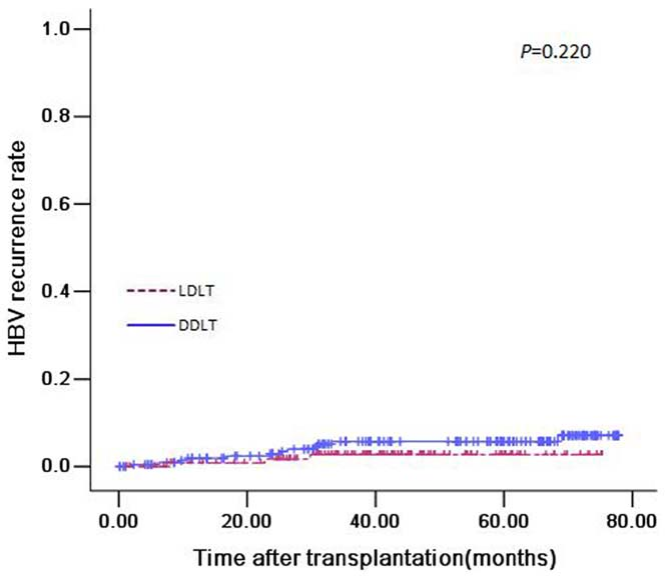
HBV recurrence curve in patients undergoing LDLT versus DDLT (*P* = 0.220).

During the follow-up period, 6 patients in the LDLT group and 9 patients in the DDLT group experienced a HBV recurrence. The 1-, 3- and 5-year HBV recurrence rates were 1.8%, 2.7% and 2.7%, respectively in the patients undergoing LDLT versus 1.4%, 5.8% and 7.1%, respectively in the patients undergoing DDLT (Figure 4). No significant difference was observed between the two groups (*P* = 0.220).

### Long-term survival between the two groups

**Figure 5:**
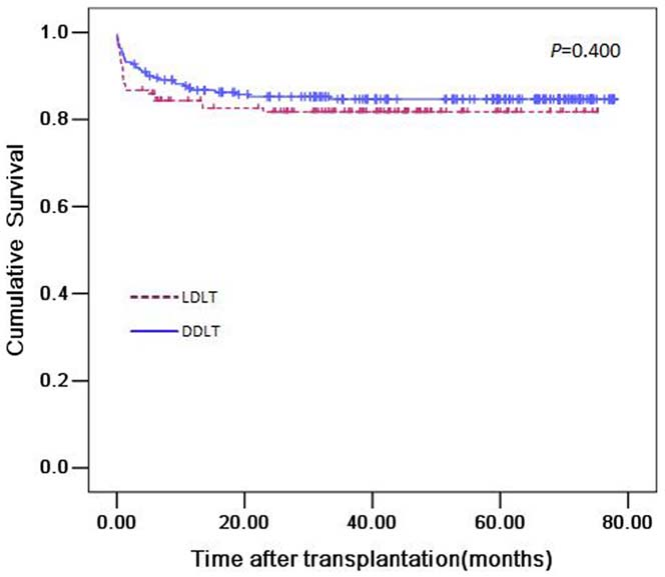
Survival curves of patients undergoing LDLT versus DDLT (*P* = 0.400).

A total of 56 (16.05%) patients died during the follow-up period. The 1-, 3- and 5-year survival rates for the LDLT and DDLT groups were 84%, 79%, 73% and 87%, 83%, 81% respectively (Figure 5). No significant difference was observed in both short-term and long-term survivals between the two groups (*P* = 0.400).

### Outcomes of emergency liver transplantations of the two groups

**Figure 6:**
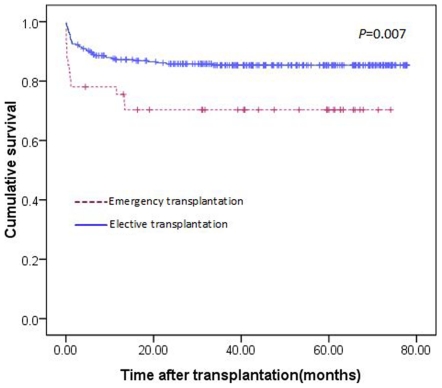
Survival curve of patients undergoing emergency liver transplantation versus elective liver transplantation (*P* = 0.007).

**Figure 7:**
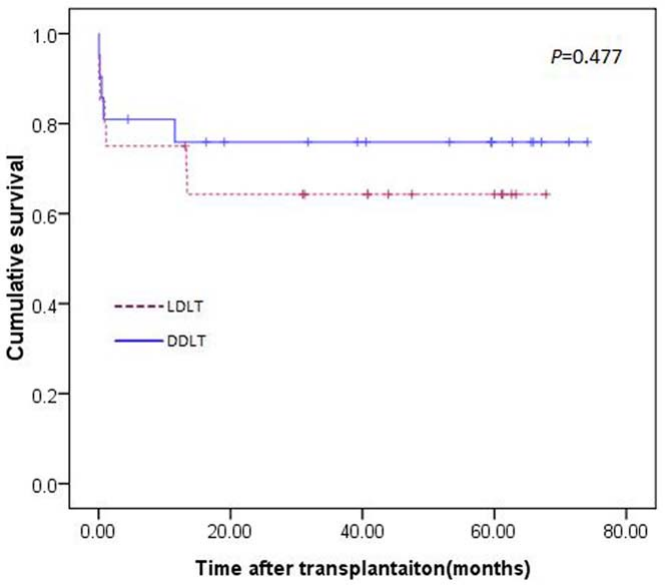
Survival curve of patients undergoing emergency liver transplantation (*P* = 0.477).

A total of 41 patients underwent emergency liver transplantations in our centre. Twenty patients received LDLT, and twenty-one patients underwent DDLT. The overall 1-, 3- and 5-year survival rates of all the patients who received emergency liver transplantations were lower than patients who underwent elective liver transplantation (75%, 68% and 63% in the emergency liver transplantation group versus 87%, 84% and 80% in the elective liver transplantation group; *P* = 0.007; Figure 6). The preoperative characteristics of patients who received emergency liver transplantation between the two groups were similar (Table 5). Among all of the emergency liver transplantation cases, the 1-, 3- and 5-year survival rates of patients who underwent LDLT versus patients who underwent DDLT were 76%, 74% and 69% versus 75%, 62% and 58%. However, this difference did not reach significant (*P* = 0.477; Figure 7).

**Table 5:** Preoperative characteristics of patients who received emergency liver transplantation.

Variables	LDLT	DDLT	*P*
**Donor variables**			
Age (year)	31.50±6.42	33.76±9.12	0.351
Gender (female)	7	4	0.310
BMI	23.21±3.18	21.87±2.64	0.139
**Recipient variables**			
Age (year)	41.55±7.85	39.67±7.51	0.437
Gender (female)	3	2	0.663
BMI	22.28±3.20	21.39±2.49	0.326
MELD score	31.00±10.99	29.81±13.65	0.761
Child-Pugh score	9.80±1.77	9.57±2.79	0.757
Starting TB level (µmol/L)	372±286	459±278	0.333
Starting Crea level (µmol/L)	124±76.60	89.64±50.42	0.095
Starting INR level	2.99±3.13	2.38±1.83	0.416

## Discussion

LDLT widely considered an alternative for DDLT. However, this treatment involves a healthy donor. It was reported that the mortality and morbidity rates of a right hepatic lobe donation are 20%–60% and 0.4%–0.6%, respectively.[20] It is important to clarify whether the outcomes for LDLT are different than DDLT. Unfortunately, few studies address this topic. In this study, we report that LDLT and DDLT have equivalent long-term survival rates, similar severe postoperative complications, similar HBV recurrence rates and required similar numbers of RBC transfusion units. Furthermore, although the incidence of immediate biliary complication in the patients undergoing LDLT was higher than those who received DDLT, the total biliary complication rate was not observed to be significantly different during long-term follow-up. Apart from the similar biliary complication rate, the incidences of postoperative vascular complications were also similar between LDLT and DDLT.

The mean waiting time for DDLT was 35.81±29.18 days, which was substantially shorter than previous reports.[21], [22] In mainland China, health insurance does not cover liver transplantation.[23], [24] Thus the cost of this surgical procedure must be paid out-of-pocket by the patient himself. This cost is a substantial financial burden for many families. Therefore many patients decline a undergoing a liver transplantation operation because of the financial burden. This concern may be the potential explanation for the shorter waiting time for DDLT. However, we believe this situation will be improved following our medical reform.

Postoperative biliary complication is commonly referred to as the “Achilles heel” of liver transplantation.[25] Although recent advances in surgical procedures and graft preservation techniques have improved outcomes for biliary reconstruction of liver transplantation, biliary complications continue to be a major cause of morbidity among liver transplant recipients and are as high as 10%–60%.[26], [27], [28] Compared with previous investigations, the incidence of postoperative biliary complication was lower in our study. This may benefit from microsurgical techniques and fixing operator in our practice.[29] Although the immediate biliary complication rate was higher in LDLT patients, the incidence of postoperative biliary complication was similar between LDLT and DDLT during the long-term follow-up period. One potential reason for this finding is that the CIT was much longer in the patients undergoing DDLT. However, a number of investigations have confirmed a long CIT may be related to late biliary complications [18], [30]. Another potential reason is that previous studies may have only taken early complications into consideration and did not have a longer follow-up period. Finally, a third potential reason is that the donor in the LDLT group was a close relative to the recipient, which may contribute to better genetic similarities and a lower acute cellular rejection rate. Liu et al. [31] confirmed the incidence of acute cellular rejection and reported that the high-degree of acute cellular rejection decreased in LDLT recipients compared to DDLT recipients. All of these may improve the outcomes. This finding indicates LDLT may have advantages when patients are followed-up for a long period. Additionally, the type of biliary complication was different between LDLT and DDLT patients. Bile leak was the most common biliary problem in patients who underwent LDLT, whereas biliary stricture was the most frequent biliary complication among patients who underwent DDLT. This result was consistent with Duailibi et al.’s findings.[32] Based on this finding, we hypothesise that surgical technique may play a critical role in biliary complications of LDLT, whereas biliary complication of DDLT is contingent upon non-surgical factors. However, further studies need to be conducted to investigate this hypothesis.

Vascular complications were another common cause of morbidity of liver transplantation, especially hepatic artery problems. The literature reports the hepatic artery complication rate to be approximately 5%–16%.[33], [34], [35] Due to the smaller vessel diameter, the insufficient length for reconstruction and the greater risk of a twist of the vascular pedicle, LDLT patients may suffer from a higher incidence of vascular complications.[36] Compared with previous investigations,[35] the hepatic artery complication rate was much lower in the present study. Additionally, LDLT and DDLT patients achieved similar outcomes in vascular reconstruction. In our practice, we reconstructed the hepatic artery using microsurgical techniques with the help of a trained vascular surgeon. During the hepatic artery reconstruction, we emphasised selecting an appropriate anastomotic artery for hepatic artery reconstruction.[37] This approach greatly reduced the hepatic artery complication rate in our transplant activity. Furthermore, intraoperative Doppler ultrasound was used in LDLT.[38] Someda et al.[39] suggests the use of intraoperative Doppler ultrasound can reduce vascular complications following liver transplantation.

A number of studies have suggested the postoperative complication rates after LDLT are higher compared to DDLT.[8], [9] However, the different early severe postoperative complication rates between LDLT and DDLT in the present study did not reach significance. According to the Clavien-Dindo classification, most Clavien I complications were trivial. Some Clavien II complications were not life-threatening.[19] The present study only compared complications ≥ grade III. Thus some complications were not considered in comparing severe early complication, such as infection, rejection and minor bile leak. This may be an explanation why the postoperative complication rate between the two groups was similar in our study. However, this finding supports why LDLT achieved similar short-term survival rates compared to DDLT. The incidence of severe early complication was lower than some previous investigations.[40] In our centre, the donors and recipients were both from the Chinese Han population. A homogenous population may have contributed to better donor-recipient compatibility. Moreover, the BMI in the Asian population is much lower than the western population.

Few investigations discuss the difference of intraoperative blood loss and the needed number of RBC transfusion units of patients with benign liver diseases undergoing LDLT or DDLT. Patients who underwent LDLT had significantly higher intraoperative blood loss than those undergoing DDLT. This difference may be due to the additional transection and the longer surgical duration of the LDLT procedure.[41] However, due to the utility of autologous blood transfusions for patients with benign liver diseases, the total allogenic RBC transfusion was similar between the two groups. In Frasco et al.'s study [42], LDLT recipients received fewer units of RBC transfusions compared to DDLT recipients. We suggest this difference may be related to the lower MELD score in the living donor transplant patients.[42]

Functional recovery is an important part of liver transplantation. Compared with DDLT, patients undergoing LDLT have similar recovery of their liver and renal functions. However, the coagulation function of patients who underwent LDLT was worse during the early postoperative days than of patients who underwent DDLT. More intraoperative blood loss and longer surgical durations may be potential explanations for this finding.[43] Nevertheless, similar liver and renal function recovery between the two groups may be the reason behind the similar postoperative complication incidence and similar long-term survival rates.

The long-term survival rates of patients undergoing LDLT versus DDLT were similar in our study. This result benefited from the similar severe early complications, equal vascular and biliary complication rate, lower HBV recurrence and similar numbers of intraoperative RBC transfusion units. However, Thuluvath et al. [10] suggested LDLT may achieve similar short-term outcomes compared with DDLT. However, the graft survival rate was significantly lower in patients undergoing LDLT. Kashyap et al. [44] reported a higher recurrence rate of primary sclerosing cholangitis in patients undergoing LDLT. This difference may be due to the difference of aetiology of the disease. The advantage of LDLT is the reduced CIT and better donor-recipient compatibility. These advantages may positively affect the long-term survival of LDLT. Moreover, Austin et al. [45] also reports the long-term survival rate in the paediatric population is better with LDLT than DDLT.

Emergency liver transplantation is a life-saving treatment for extremely sick patients. However, different countries or centres have different criteria for emergency liver transplantation.[12], [46], [47], [48], [49], [50] In our country, different provinces have different criteria. Consistent with previous studies, the long-term survival rate of patients undergoing emergency liver transplantation was lower than of patients undergoing elective transplantation.[51] The outcomes of patients undergoing emergency LDLT and undergoing emergency DDLT were similar. This result may suggest that for sicker patients, LDLT may achieve similar outcomes to DDLT. However, Mark et al. [52] reported the 3-month and 6-month postoperative survival rates were substantially better in the LDLT group than the DDLT group in the paediatric population in emergent situations. They attributed this difference to shorter waiting times in the LDLT group, which may prevent disease progression. However, as shown in table 5, the preoperative variables of the two groups were comparable in the present study. This may be why outcomes after emergency LDLT and emergency DDLT were similar in the present study.

In our study, we excluded patients with malignant liver diseases to eliminate the negative influences of tumour recurrence as a late complication. However, there are also some limitations in our study. We did not compare the incidence of rejection between the two groups. Although previous investigations report a lower rejection rate for LDLT than DDLT in the paediatric and adult patient populations,[31], [53] this topic is still controversial and deserves further study. Additionally, the mean follow-up period of the LDLT group was shorter than the DDLT group. This was because of more DDLTs were performed in 2005. In 2005, we performed 103 liver transplantations for patients with benign end-stage liver disease, including 18 LDLTs and 85 DDLTs. The proportion of LDLT increased in the later transplant period. Although the conclusions we report include all liver transplantations from 2005 to 2011 for patients with benign end-stage liver disease, the mean follow-up time specifically for the LDLT group is much shorter than the DDLT group. We suggest that many postoperative complications occurred in the early postoperative period. For instance, the late biliary complications (> 3 postoperative months) occurred from 4 to 26 postoperative months. This was supported by the findings from the paediatric liver transplantation group. Berrocal et al. [54] reported that most vascular and biliary complications after paediatric liver transplantation occur in the early postoperative period, especially the first 3 postoperative months. However, the mean follow-up period for the LDLT group was 34.58±21.53 months. We thus believe the difference in the length of the follow-up period may not be very influential in the final conclusion.

In conclusion, we report there is a role for LDLT for patients with benign liver diseases. Patients undergoing LDLT have similar outcomes to patients undergoing DDLT. Specifically, outcomes include a similar incidence of severe postoperative complications, a vascular complication rate, HBV recurrence rate and long-term survival rate. Emergency LDLT can achieve similar long-term survival rates to emergency DDLT. Additionally, similar biliary complication rates between LDLT and DDLT during a long-term follow-up period was observed, although it was noted that patients who underwent LDLT may suffer from a higher incidence of immediate biliary complication.
